# Supplementary figures and images for: Profiling and initial validation of urinary microRNAs as biomarkers in IgA nephropathy
Source: PeerJ. 2015 Jun 2;3:e990. doi: 10.7717/peerj.990 (PMC4458130; doi:10.7717/peerj.990)

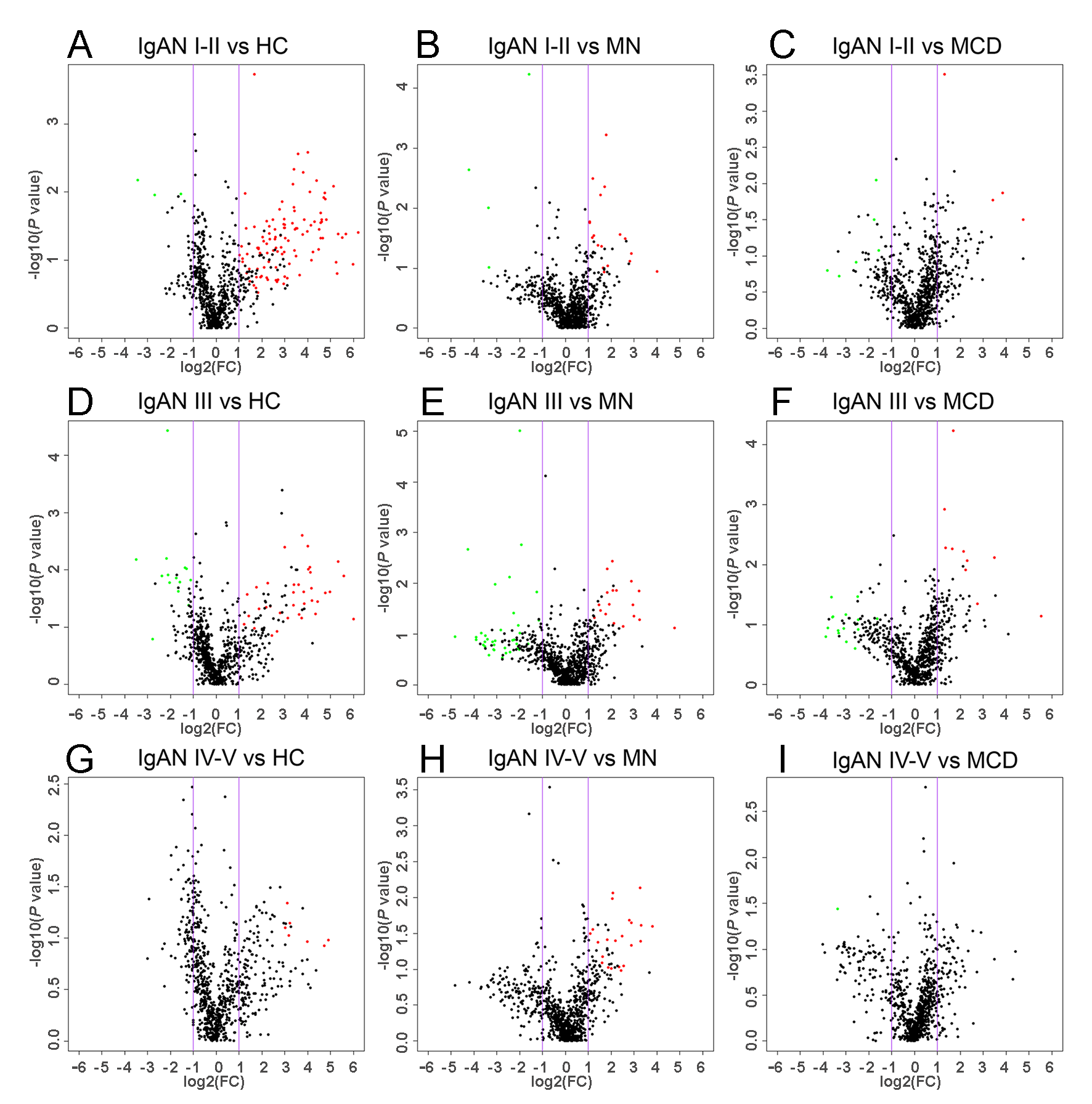

Supplement: Figure S1 — Urinary miRNAs levels were analyzed by miRNA microarray in the screening cohort, which including 6 patients with IgAN grade I–II, 6 patients with IgAN grade III, 6 patients with IgAN grade IV–V, 4 patients with MN, 4 patients with MCD and 6 healthy controls. Red and green dots represent the number of miRNAs that were significantly up-regulated and down-regulated, respectively, and black dots represent a lack of differential expression. Thethreshold of statistically significant difference was set at P < 0.05 and FC > 2. IgAN, IgA nephropathy; I–II, III and IV–V, grade I–II, III and IV–V according to Lee’s grading system, respectivley; HC, healthy control; MN, membranous nephropathy; MCD, minimal change disease; FC, fold change. [file peerj-03-990-s001.png]
