# Supplementary material for: Profiling and initial validation of urinary microRNAs as biomarkers in IgA nephropathy
Source: PeerJ. 2015 Jun 2;3:e990. doi: 10.7717/peerj.990 (PMC4458130; doi:10.7717/peerj.990)
Supplement: Supplemental Information 1 [file peerj-03-990-s007.docx]

**Additional information regarding the qPCR analysis procedure**

Information of the performed qPCR analysis of urinary miRNA expression based on the MIQE (Minimum Information for Publication of Quantitative Real-Time PCR Experiments) guidelines. All essential information ( E ) must be submitted with the manuscript. Desirable information ( D ) should be submitted if available.

**Experimental design**

**Definition of experimental and control groups ( E )**

We analysed urine samples from patients with IgA nephropathy (IgAN). Control groups consisted of healthy subjects as healthy control (HC) group, patients with membranous nephropathy (MN) as MN group and patients with minimal change disease (MCD) as MCD group.

**Number within each group ( E )**

The screening cohort: IgAN n=18; HC n=6; MN n=4; MCD n=4.

The validation cohort: IgAN n=102; HC n=34; MN n=41; MCD n=27.

**Assay carried out by the core or investigator’s laboratory? ( D )**

By the investigator’s laboratory.

**Sample**

**Description ( E )**

A whole-stream early morning urine specimen was collected at the day of renal biopsy.

**Volume/mass of sample processed ( D )**

We used a whole-stream early morning urine for RNA purification.

**Microdissection or macrodissection ( E )**

No dissection.

**Processing procedure ( E )**

The urine sample was centrifuged at 3,000 g for 30 min at 4°C and the supernatant was discarded. Then the sample was centrifuged at 13,000 g for 5 min at 4°C and the supernatant was discarded again. The urinary sediments were lysed by adding 1 ml Buffer MZ and mixed thoroughly by pipetting up and down several times. Place the tube containing the homogenate on the benchtop at room temperature (15-25°C) for 5 min and then stored in eppendorf tubes at -80°C.

**If frozen, how and how quickly? ( E )**

Sample procession was performed within 180 minutes. Once the urinary sediments were lysed by Buffer MZ (Tiangen Biotech, Beijing, China, Catalog number DP501 ), they were immediately stored at –80°C until used.

**If fixed, with what and how quickly? ( E )**

No fixation.

**Sample storage conditions and duration ( E )**

Samples were stored at –80°C and used within three months.

**Nucleic acid extraction**

**Procedure and/or instrumentation ( E )**

1. The urinary sediment lysed by Buffer MZ was thawed on ice.

2. Centrifuge the lysate at 12,000 rpm for 5 min at 4°C. Then transfer supernatant to a new tube.

3. Add 200 μl chloroform to the supernatant and cap it securely. Shake the tube vigorously for 15 s, put the tube containing the homogenate on the bench top at room temperature (15 -25°C) for 5 min.

4. Centrifuge for 15 min at 12,000 rpm at 4°C. After centrifugation, the sample separates into 3 phases: an upper, colorless, aqueous phase containing RNA; a white interphase; and a lower organic phase. The volume of aqueous phase is around 50% of Buffer MZ added, transfer the aqueous phase to a new tube.

5. Add 1.5 volume of 100% Ethanol and mix thoroughly by pipetting up and down several times. Transfer the obtained liquid, include any precipitate that may have formed into a Spin Column miRspin, centrifuge at 12,000 rpm for 30 s at room temperature (15-25°C). Discard the flow-through, and keep the Spin Column miRspin after centrifugation.

6. Add 500 μl Buffer MRD to the Spin Column miRspin (ensure that 100% Ethanol has been added). Close the lid gently and incubate 2 min at room temperature (15-25°C). Then centrifuge for 30 s at 12,000 rpm to wash the column. Discard the flow-through.

7. Add 600 μl Buffer RW to the Spin Column miRspin (ensure that 100% Ethanol has been added). Close the lid gently and incubate 2 min at room temperature (15-25°C). Then centrifuge for 30 s at 12,000 rpm to wash the column. Discard the flow-through.

8. Repeat step 8.

9. Place the Spin Column miRspin into a new 2 ml collection tube, centrifuge at 12,000 rpm for 1 min, and discard the flow-through.

10. Transfer the Spin Column miRspin to a new 1.5 ml RNase-Free Centrifuge Tube, add 30-100 μl RNase-Free ddH2O directly onto the miRspin column membrane and incubate 2 min at room temperature (15-25°C). Close the lid gently and centrifuge for 2 min at 12,000 rpm to elute the RNA.

**Name of kit and details of any modifications ( E )**

The miRcute miRNA Isolation Kit (Tiangen Biotech, Beijing, China, Catalog number DP501 ) was used to purify total RNA from urine sediments. RNA isolation was performed according to the manufacturer’s recommendation.

**Source of additional reagents used ( D )**

Chloroform (Beijing Chemical Works, China, catalog number B0102004);

100% Ethanol (Beijing Chemical Works, China, catalog number B0301002).

**Details of DNase or RNase treatment ( E )**

We did not perform DNase or RNase treatment for serum samples.

**Contamination assessment (DNA or RNA) ( E )**

Contamination was assessed by a ‘no reverse transcription’ control in the qPCR reaction. Furthermore, a melting curve analysis was performed as standard in order to detect DNA contamination of the RNA which would be visible as further unspecific peak.

**Nucleic acid quantification ( E )**

RNA concentration (ng/µl) was determined spectrophotometrically using NanoDrop 2000 spectrophotometer ( Thermo Scientific, USA ).

**Instrument and method ( E )**

NanoDrop 2000 spectrophotometer( Thermo Scientific, USA )

Performed in accordance with manufacturer's recommendations.

**Purity (A260/A280) ( D )**

1.95 (range = 1.82 -2.07, SD= 0.06)

**Yield ( D )**

22.73 ng/ml (interquartile range [IQR] = 10.75 - 40.35)

**RNA integrity: method/instrument ( E )**

RNA integrity was not checked.`

**RIN/RQI or Cq of 3' and 5' transcripts ( E )**

RNA integrity was not checked.

**Inhibition testing (Cq dilutions, spike, or other) ( E )**

Inhibition testing was not performed.

**Reverse transcription**

**Complete reaction conditions ( E )**

Reverse transcription was performed with 500ng isolated RNA using the miRcute miRNA First-Strand cDNA Synthesis Kit (Tiangen Biotech, Beijing, China, catalog number KR201) according to the manufacturer’s recommendation.

1. Add Poly (A) to the 3’ terminal of miRNA

1) Prepare a reaction solution in a pre-cooling tube on ice according to the following table (Add E.coli Poly (A) Polymerase in the last step).

| Contents | Volume/Reaction | Final concentration |
| --- | --- | --- |
| Total RNA* | - | ≤2µg |
| E.coli Poly (A) Polymerase (5 U/µl) | 0.4µl | 2U |
| 10×Poly (A) Polymerase Buffer | 2µl | 1× |
| 5×rATP Solution | 4µl | 1× |
| Rnase-free H2O | - | - |
| Total volume | 20µl |  |

*Total RNA as the template has to contain miRNA in the reaction. miRNA can also be used as template in the reaction (2-5μl miRNA is recommended. Determine the amount according to the abundance of the target miRNA.)

2) Mix gently by pipetting the solution and centrifuge briefly to remove the drops from walls of tube. Incubate at 37°C for 60 min. The solution can be used directly to the downstream experiments or stored at -20 °C for a short time.

2. Reverse transcription of Poly (A) modified miRNA

1) Prepare a reaction solution according to the following table

| Contents | Volume |
| --- | --- |
| Poly (A) reaction solution | 2µl |
| 10×RT Primer | 2µl |
| 10×RT Buffer | 2µl |
| Super pure d NTP (2.5Mm each) | 1µl |
| Rnasin (40U/µl) | 1µl |
| Quant RTase | 0.5µl |
| Rnase-free ddH2O | 11.5µl |
| Total volume | 20µl |

2) Mix gently by pipetting the reaction solution and briefly centrifuge to remove drops from the wall of the tube. Incubate at 37°C for 60 min.

The reaction solution including cDNA products can be stored in eppendorf tubes at -20°C or used in downstream quantitative PCR directly.

**Amount of RNA and reaction volume ( E )**

We used 500ng of isolated urine RNA for reverse transcription.

The reaction volume for all samples was 20 μl.

**Priming oligonucleotide (if using GSP) and concentration ( E )**

We used the miRcute miRNA First-Strand cDNA Synthesis Kit (Tiangen Biotech, Beijing, China, catalog number KR201). Priming oligonucleotide and its concentration are not provided by the manufacturer.

**Reverse transcriptase and concentration ( E )**

We used the miRcute miRNA First-Strand cDNA Synthesis Kit (Tiangen Biotech, Beijing, China, catalog number KR201). Reverse transcriptase and concentration are not provided by the manufacturer.

**Temperature and time ( E )**

60 minutes at 37°C, followed by 60 minutes at 37°C.

**Manufacturer of reagents and catalogue numbers ( D )**

miRcute miRNA First-Strand cDNA Synthesis Kit (Tiangen Biotech, Beijing, China, catalog number KR201)

**Cqs with and without reverse transcription ( D )**

For the performed qPCR studies, in ‘no reverse transcription control samples’ (RNA not treated with reverse transcription enzyme) no amplification was detected.

**Storage conditions of cDNA ( D )**

We stored cDNA at -20 °C for up to two weeks.

**qPCR target information**

**Gene symbol ( E )**

*hsa-miR-223-3p, hsa-miR-629-5p, hsa-miR-150-5p, hsa-miR-572, hsa-miR-371b-5p,* *hsa-miR-3613-3p, hsa-miR-4668-5p* and *hsa-miR-6750-5p.*

**Sequence accession number ( E )**

| Gene symbol | Sequence accession number |
| --- | --- |
| *hsa-miR-150-5p* | MIMAT0000451 |
| *hsa-miR-223-3p* | MIMAT0000280 |
| *hsa-miR-629-5p* | MIMAT0004810 |
| *hsa-miR-3613-3p* | MIMAT0017991 |
| *hsa-miR-6750-5p* | MIMAT0027400 |
| *hsa-miR-371b-5p* | MIMAT0019892 |
| *hsa-miR-572* | MIMAT0003237 |
| *hsa-miR-4668-5p* | MIMAT0019745 |

**Amplicon length ( E )**

All primers were purchased pre-designed from Tiangen Biotech; the location of the amplicon is not provided by the manufacturer.

**In silico specificity screen (BLAST, and so on) ( E )**

All primers were purchased pre-designed from Tiangen Biotech; BLAST searches were performed by the manufacturer.

**Location of each primer by exon or intron (if applicable) ( E )**

All primers were purchased pre-designed from Tiangen Biotech, and details on the location are not provided.

**What splice variants are targeted? ( E )**

All primers were purchased pre-designed from Tiangen Biotech.

**qPCR oligonucleotides**

**Primer sequences ( E )**

All primers were purchased pre-designed from Tiangen Biotech. The primer sequence is not supplied by the manufacturer.

**Probe sequences ( D )**

miRcute miRNA SYBR Green qPCR Detection kit (Tiangen Biotech, Beijing, China, catalog number FP401) does not have any probes.

**Location and identity of any modifications ( E )**

All primers were purchased pre-designed from Tiangen Biotech, and this information is not provided.

**Manufacturer of oligonucleotides ( D )**

Tiangen Biotech, Beijing, China.

**qPCR protocol**

**Complete reaction conditions ( E )**

Quantitative real-time PCR was performed using the miRcute miRNA SYBR Green qPCR Detection kit (Tiangen Biotech, Beijing, China, catalog number FP401) on an 7500 Fast Dx Real-Time PCR Instrument (Applied Biosystems, CA, USA) in triplicate (20 μl reaction volume). All experiments were performed as specified in the manufacturer’s protocols.

**Reaction volume and amount of cDNA/DNA ( E )**

We used 2 μl of cDNA in 20 μl total reaction volume of the PCR.

**Primer, (probe), Mg2+, and dNTP concentrations ( E )**

We used the miRcute miRNA SYBR Green qPCR Detection kit (Tiangen Biotech, Beijing, China, catalog number FP401). The concentration of Mg^2+^ or dNTPs in the 2×miRcute miRNA Premix is not provided.

**Polymerase identity and concentration ( E )**

We used miRcute miRNA SYBR Green qPCR Detection kit (Tiangen Biotech, Beijing, China, catalog number FP401). It includes the antibody modified DNA Polymerase. Its concentration is not provided by the manufacturer.

**Buffer/kit identity and manufacturer ( E )**

miRcute miRNA SYBR Green qPCR Detection kit (Tiangen Biotech, Beijing, China, catalog number FP401).

**Additives (SYBR Green I, DMSO, and so forth) ( E )**

SYBR Green I is included in the 2×miRcute miRNA Premix.

**Manufacturer of plates/tubes and catalog number ( D )**

Tubes library 8-strip RK sterile (Labcon, USA, catalogue number 3911-545-000)

**Complete thermocycling parameters ( E )**

The thermo cycling conditions were, as specified in the manufacturer’s protocol: 2 minutes at 94°C, followed by 40 cycles of: 20 seconds at 94°C, 34 seconds at 60°C. Subsequently we performed melting curve analyses to confirm specificity of the PCR products.

**Reaction setup (manual/robotic) ( D )**

The qPCR was setup up manually.

**Manufacturer of qPCR instrument ( E )**

7500 Fast Dx Real-Time PCR Instrument (Applied Biosystems, CA, USA)

**qPCR validation**

**Specificity (gel, sequence, melt, or digest) ( E )**

The specificity of the amplification products have been confirmed by analyzing their melting curves.

**For SYBR Green I, Cq of the NTC ( E )**

No signal detected in NTCs, Cq of the NTCs undetermined or higher than 38

**Calibration curves with slope and y intercept ( E )**

| Gene symbol | Slope | Y intercept |
| --- | --- | --- |
| *hsa-miR-150-5p* | -3.275 | 34.93 |
| *hsa-miR-223-3p* | -3.428 | 33.23 |
| *hsa-miR-629-5p* | -2.873 | 35.54 |
| *hsa-miR-3613-3p* | 3.365- | 30.80 |
| *hsa-miR-6750-5p* | -3.534 | 37.16 |
| *hsa-miR-371b-5p* | -3.417 | 36.52 |
| *hsa-miR-572* | -3.302 | 35.93 |
| *hsa-miR-4668-5p* | -3.042 | 38.37 |

**PCR efficiency calculated from slope ( E )**

*hsa-miR-150-5p*: 102%; *hsa-miR-223-3p*: 96%; *hsa-miR-629-5p*, 123%; *hsa-miR-3613-3p*, 98%; *hsa-miR-6750-5p*, 92%; *hsa-miR-371b-5p*, 96%; *hsa-miR-572*, 101%; *hsa-miR-4668-5p*, 113%.

**r2 of calibration curve ( E )**

*hsa-miR-150-5p*: 0.989; *hsa-miR-223-3p*: 0.988; *hsa-miR-629-5p*, 0.993; *hsa-miR-3613-3p*, 0.995; *hsa-miR-6750-5p*, 0.924; *hsa-miR-371b-5p*, 0.994; *hsa-miR-572*, 0.985; *hsa-miR-4668-5p*, 0.957.

**Linear dynamic range ( E )**

In the tested range, reactions were linear from 10^1^ to 10^7^ genome copies/well, and had potential to cover wider range at higher orders of magnitude.

**Cq variation at LOD ( E )**

All primers were pre-designed; the performance is guaranteed by the manufacturer. 0.4 - 0.7 cycle

**Evidence for LOD ( E )**

All primers were pre-designed; the performance is guaranteed by the manufacturer. No amplification after 38 cycles

**If multiplex, efficiency and LOD of each assay ( E )**

We did not perform multiplex assays.

**Data analysis**

**qPCR analysis program (source, version) ( E )**

The analysis of the real-time PCR data was done using the SDS software v2.4 (settings: automatic baseline, threshold 0.2); relative microRNA levels were calculated with the RQ Manager v1.2.1, and data was analyzed with DataAssist v2.0 (all software packages: Applied Biosystems).

Instrument compliant 7500 software ( Applied Biosystems, USA, version 2.0.5 );

Microsoft Office Excel 2013 ( Microsoft, USA, version 2013).

**Method of Cq determination ( E )**

The relative quantification of microRNA expression was performed using the comparative CT (ΔΔCT) method using the Data Assist v2.0 software.

**Outlier identification and disposition ( E )**

By analyzing the real-time PCR data using the SDS software v2.4 with the following settings: automatic baseline, threshold 0.2, outliers were identified and erased.

**Results for NTCs ( E )**

No signal detected in NTCs. Cq of the NTCs undetermined or higher than 38.

**Justification of number and choice of reference genes ( E )**

Not applied.

**Description of normalization method ( E )**

Endogenous reference gene.

**Number and concordance of biological replicates ( D )**

The number of biological replicates was from 4 to 102.

**Number and stage (reverse transcription or qPCR) of technical replicates ( E )**

Each biological replicate was run in triplicate.

**Repeatability (intraassay variation) ( E )**

On a average within 0.25 cycle with a maximum of 0.5-1 cycles for all samples.

**Statistical methods for results significance ( E )**

microRNA levels in different groups were analyzed using the Mann- Whitney-U or Kruskal-Wallis-test, as appropriate. Clinicalpathological parameters and microRNA levels were correlated using the Spearman analysis.

**Software (source, version) ( E )**

Statistical analyses were performed using IBM SPSS Statistics v20 (IBM, Ehningen, Germany) and Microsoft Office Excel 2013 ( MicroSoft, USA, version 2013).
