# Supplementary material for: Profiling and initial validation of urinary microRNAs as biomarkers in IgA nephropathy
Source: PeerJ. 2015 Jun 2;3:e990. doi: 10.7717/peerj.990 (PMC4458130; doi:10.7717/peerj.990)
Supplement: Supplemental Information 2 [file peerj-03-990-s008.pdf]

知情同意书

尊敬的患者：

我们邀请您参加一项由中国人民解放军总医院肾脏病国家重点学科、肾脏疾病国家重点实验室主持、国家高技术研究发展计划（863 计划）课题《IgA 肾脏疾病分子分型和个体化诊疗技术》研究。本研究已经得到中国人民解放军总医院医学伦理委员会的审查和批准。本文涵盖的部分内容由法规要求而定，并且为了保护参加研究的患者的权益，本文经伦理委员会审核并同意。在您同意参加前，请您阅读并理解以下资料，更好地了解该研究的有关信息，以决定是否参加这项研究。

为什么要开展本项研究？

研究背景：流行病学调查证据表明，IgA 肾病是最为常见的原发性肾小球肾炎，是导致终末期肾脏疾病（尿毒症）的最主要病因，因此，有效诊治 IgA 肾病，是减少发展至尿毒症的关键环节。目前对 IgA 肾病的诊断仍依赖肾活检，但是肾活检是有创检查，不能经常、反复做，而多数 IgA 肾病是进展性疾病，因此，需要能及时反映 IgA 肾病病情变化的生物标志物。

研究的目的：本研究在已经建立的全国多中心合作研究的 IgA 肾病协作网的良好工作平台的基础上，进一步扩大、完善 IgA 肾病的临床、病理、生物信息数据库和样本资源库。在此基础上结合肾脏病理和尿分子标志物的检测，发现评价 IgA 肾病的新的生物标志物及分子分型，指导患者预后评估和个体化治疗，提高 IgA 肾病的临床治疗效果。

研究概况

如果您同意参加本次研究，并且在知情同意书中签名表示同意，在不影响您身体康复的前提下，我们将您的临床信息录入数据库，并收集您肾穿当天晨尿以备研究使用。

研究程序

本研究以同意参加本研究并签署本知情同意书为零时间点，将持续 24 月共

8 次访视，在此期间，您需要来医院做一些检查、按日程进行回访，并告诉我们您的任何变化。研究内容包括：①一般资料及常规临床指标采集：一般资料包括：年龄、性别、血压、体重指数；血检验指标包括：血红蛋白、血肌酐、血尿素氮、血尿酸、血糖、甘油三脂、胆固醇、总蛋白、白蛋白；尿检验指标包括：尿常规、24 小时尿蛋白定量。②病理指标的采集：为肾穿时的病理标本，由病理医师进行病理指标的评价，最后综合评分，从而保证病理指标的严谨性和可重复性。

其他的治疗选择

参加本研究可能改善或不能改善您的健康状况，您可以选择:

- 不参加本研究，继续您的常规治疗。
- 参加别的研究。
- 不接受任何治疗。

请与您的医生协商您的决定。

该研究可能会带来的影响

如果您有关于研究中检查和步骤的任何疑问可以向研究医生咨询。

研究的风险和不良反应

我们收集尿液样本的方法与临床常规检查相同，不会对您的健康造成影响。如果您在访视之间出现任何与本研究相关或不相关的不适，请及时给您的研究医生打电话咨询。

您需告诉您的家人或与您亲近的朋友您正在参加一项临床研究，他们可以注意上面描述的事件。如果他们对您参加研究有疑问，您可以告诉他们怎样联系您的研究医生。

研究获益

从本研究中得到的信息将有助于确定哪种治疗方法可以更安全有效地诊断、治疗与您患有相似病情的其他患者。研究结果可能促进新药物和新医疗产品的产生，对改善未来病人的治疗有所帮助。

报酬或补偿

您参与本研究后，我们将对您的病情进行随访，为您的治疗提供及时咨询帮助。我们利用您样本的研究结果可能对评估您将来的病情变化及改善未来病人的诊治有所帮助。尽管您为本研究做出了贡献，但您没有直接的经济补偿。研究所致伤害的医疗费用赔偿如果您的健康确因参加这项研究而发生与研究相关的损害，请立即通知研究医生，研究医生将负责对您采取适当的治疗措施。即使您已经签署这份知情同意书，您仍然保留您所有的合法权利。

保密性

您的所有个人资料和观察记录均属保密，仅供本研究使用。除非法律另有规定，否则只有研究人员、伦理委员会以及在特定情况下的有关政府部门才可以查看有您姓名的病例记录。本次研究收集的数据和信息可能会发表，但有关本项研究结果的公开报告将不会披露您的个人身份。我们将在法律允许的范围内，尽一切努力保护您个人医疗资料的隐私。关于您的个人和医疗信息将对外保密，且被保管在安全可靠的地方。在任何时候，您可以要求查阅您的个人信息（比如您的姓名和地址），如有需要可以修改这些信息。我们可能将您的样本与合作者进行研究，但我们保证不会提供您的姓名、电话、地址等信息，而是以标本编号的方式来进行。我们不向您的医生报告研究结果，您的检测结果也将不记录在病历当中。您的样本将在有效期之内保存使用，有效期之后将销毁处理。在有效期之内，如果您不希望自己的样本被使用，您随时可以与我们联系，中止对您样本的研究，我们将会对它进行销毁处理。当您签署了这份知情同意书，代表您同意您的个人和医疗信息被用于上述所描述的场合。

自愿参加

参加本研究是完全自愿的，您可以拒绝参加研究，或者研究过程中的任何时候选择退出研究，不需任何理由。该决定不会影响您未来的治疗。如果您不参加本研究，或中途退出研究，您的临床信息数据将被从数据库中清除，样本将被销毁。如果您决定退出本研究，请提前通知您的研究医生。为了保障您的安全，您可能被要求进行相关检查，这对保护您的健康是有利的。

同意签字页

课题名称：IgA 肾脏疾病分子分型和个体化诊疗技术

受试者同意声明：

我已经仔细阅读了上述有关本研究的介绍，并且深刻理解该研究的目的、研究程序以及参加该项研究带来的益处和可能的风险，我的医生已经向我作了详细说明，对所有相关问题均已得到令我满意的解答，确认已有充足的时间进行考虑。我自愿签署这份知情同意书，并自愿参加本文所介绍的临床研究，我已同时收到一份有签名并注明日期的该知情同意书副本。我可以随时退出本研究，医疗待遇与权益不会受到影响。

我同意____ 不同意____ 除本研究以外的其他研究经过伦理委员会批准后可以利用我的医疗记录和病理检查标本。

受试者签名________ 日期________

受试者姓名正楷________

受试者联系电话________

法定代理人签名________ 日期________

法定代理人姓名正楷________

研究者声明：

我确认已向患者解释了本研究的详细情况，特别是参加本研究可能产生的风险和收益。

研究者签名________ 日期________

研究者姓名正楷________

研究者联系电话________
